# Correction: Kobori et al. Utilization of Corn Steep Liquor for the Production of Fairy Chemicals by *Lepista sordida* Mycelia. *J. Fungi* 2022, *8*, 1269

**DOI:** 10.3390/jof10110744

**Published:** 2024-10-29

**Authors:** Hajime Kobori, Jing Wu, Hirohide Takemura, Jae-Hoon Choi, Naoto Tada, Hirokazu Kawagishi

**Affiliations:** 1 Iwade Research Institute of Mycology Co., Ltd., 1-9 Suehiro, Tsu 514-0012, Japan; 2 Research Institute for Mushroom Science, Shizuoka University, 836 Ohya, Suruga-ku, Shizuoka 422-8529, Japan; 3 Faculty of Agriculture, Shizuoka University, 836 Ohya, Suruga-ku, Shizuoka 422-8529, Japan; 4 Graduate School of Science and Technology, Shizuoka University, 836 Ohya, Suruga-ku, Shizuoka 422-8529, Japan; 5 Research Institute of Green Science and Technology, Shizuoka University, 836 Ohya, Suruga-ku, Shizuoka 422-8529, Japan

## Error in Figures

In the original publication [1], there were errors in Figures 2, 4, 6 and 8. The units on the X-axis of those graphs were in mg/mL, but the correct units were μg/mL. Therefore, the units of the X-axis in these four figures have been revised from mg/mL to μg/mL, as shown in the Figure 2, Figure 4, Figure 6 and Figure 8 below.

## Figures and Tables

**Figure 2:**
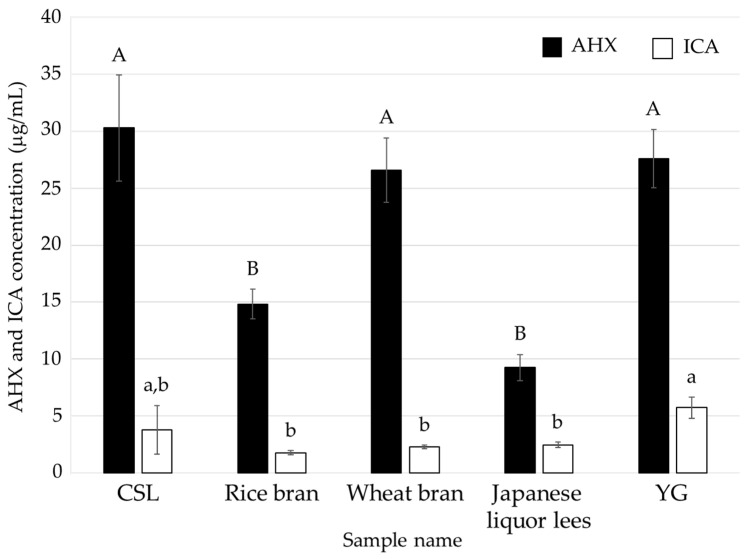
AHX and ICA contents of the culture filtrates of *L. sordida* mycelia cultured in media containing a food industrial by-product or YG. Different alphabet letters (capital letters for AHX, and small letters for ICA) indicate significant differences (Tukey–Kramer post hoc test, *p* < 0.05; *n* = 3).

**Figure 4:**
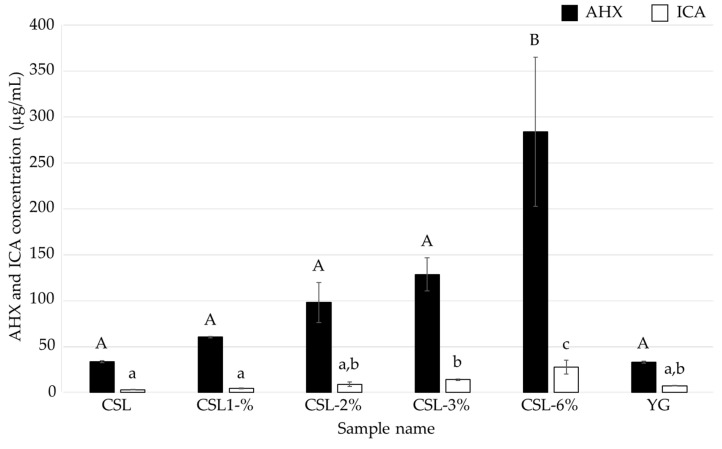
AHX and ICA contents of the culture filtrates of *L. sordida* mycelia cultured in media containing various concentrations of CSL. Different alphabet letters (capital letters for AHX, and small letters for ICA) indicate significant differences (Tukey–Kramer post hoc test, *p* < 0.05; *n* = 3).

**Figure 6:**
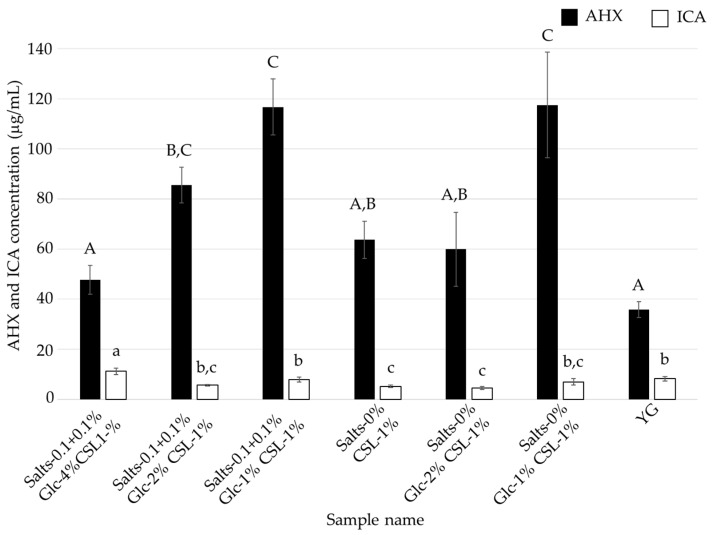
AHX and ICA contents of the culture filtrates of *L. sordida* mycelia cultured in CSL-1% media with reduced concentrations of glucose and inorganic salts. Different alphabet letters (capital letters for AHX, and small letters for ICA) indicate significant differences (Tukey–Kramer post hoc test, *p* < 0.05; *n* = 3).

**Figure 8:**
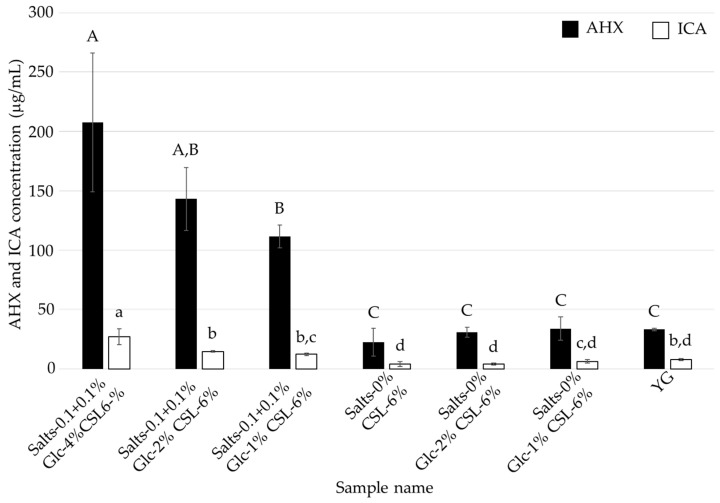
AHX and ICA contents of the culture filtrates of *L. sordida* mycelia cultured in CSL-6% media with reduced concentrations of glucose and inorganic salts. Different alphabet letters (capital letters for AHX, and small letters for ICA) indicate significant differences (Tukey–Kramer post hoc test, *p* < 0.05; *n* = 3).
